# Supplementary material for: Prevalence and Predictors of Antibiotic Self-Medication in Sudan: A Descriptive Cross-Sectional Study
Source: Antibiotics (Basel). 2023 Mar 20;12(3):612. doi: 10.3390/antibiotics12030612 (PMC10045343; doi:10.3390/antibiotics12030612)
Supplement: Supplementary file 1 [file antibiotics-12-00612-s001.zip › antibiotics-2197887-supplementary.pdf]

Supplementary file- Table S1

Stratification of the sample based on states:

States	Frequency	Percentage (%)
Northern	92	6.2
River Nile	82	5.5
Red Sea	86	5.8
Kassala	79	5.3
Gedaref	83	5.6
El Gazira	76	5.1
Blue Nile	95	6.4
Sennar	76	5.1
White Nile	84	5.6
North Kordofan	91	6.1
South Kordofan	90	6.0
West Kordofan	64	4.3
East Darfur	90	6.0
South Darfur	80	5.4
Central Darfur	66	4.4
North Darfur	82	5.5
West Darfur	81	5.4
Khartoum	95	6.4
**Total**	**1492**	**100.0**

Supplementary file- Table S2

QUESTIONNAIRE FOR DATA COLLECTION

Prevalence and predictors of antibiotic self-medication in Sudan: A descriptive cross-sectional study.

General information (filled by instructors and/or data collectors)

Data of interview:……../……./………

Participants ID No: ………………………………………………

**Section one: Demographical characteristics**			
No	Questions	Response	Coding
1.	Age	A. 18-24<br>B. 25-39<br>C. 40-59<br>D. More than 60	
2.	Gender	A. male<br>B. female	
3.	Educational level	A. primary school<br>B. high school<br>C. graduated<br>D. post graduated	
4.	Monthly income	A. less than 12500<br>B. 12500-25000<br>C. 25000-37500<br>D. More than 37500	
5.	Health insurance	A. Yes<br>B. No	

**Section two: reasons, factors, and practice of self-medication with antibiotics.**			
No	Questions	Responses	code
1.	In the last 12 months have you ever taken antibiotics to treat yourself without a prescription from a health professional?	A. Yes<br>B. No	
2.	What was (were) your reasons of self-medication with antibiotic (check more than if needed)	A. cost saving<br>B. convenience<br>C. lack of trust in prescribing doctor	
3.	For which of the following complaint(s) did you use antibiotics (check more than one if needed)	A. Tonsillitis<br>B. Cough<br>C. Runny nose<br>D. Nasal congestion<br>E. Fever<br>F. Pain<br>G. Diarrhoea<br>H. Wound infection<br>I. Vomiting	
4.	Your selection of antibiotics was based on… (check more than one if applicable)	A. Recommendation by community pharmacists<br>B. Opinion of family members<br>C. Opinion of friends<br>D. My own experience<br>E. Previous doctor's prescription	
5.	What did you consider when selecting antibiotics? (check more than one if applicable)	A. Type of antibiotics<br>B. Brand of antibiotics<br>C. Price of antibiotics<br>D. Indications for use	
6.	Where did you usually obtain antibiotics from for self-medication? (check more than one if applicable)	A. Community pharmacies<br>B. Leftover from previous prescription	
7.	Did you ever check the instructions come with the package insert of antibiotics for self-treatment?	A. Yes, always<br>B. Yes, sometimes<br>C. Never<br>If Never, please go to Question 9	
8.	How much did you understand the instructions?	A. Fully understood<br>B. Partly understood	

		C. Did not understand at all	
9.	How did you know the dosage of antibiotics? (check more than one if applicable)	A. By checking the package insert<br><br>B. By consulting a doctor<br><br>C. By consulting a pharmacist<br><br>D. By consulting family members/friends<br><br>E. From the Internet<br><br>F. From my previous experience<br><br>G. By guessing the dosage by myself	
10.	Did you ever change the dosage of antibiotics deliberately during the course of self-treatment?	A. Yes, always<br><br>B. Yes, sometimes<br><br>C. Never<br><br>If Never, please go to Question 12	
11.	Why did you change the dosage of antibiotics during the course of self-treatment? (check more than one if applicable)	A. Improving conditions<br><br>B. Worsening conditions<br><br>C. To reduce adverse reactions<br><br>D. Drug insufficient for complete treatment	
12.	Did you ever switch antibiotics during the course of self-treatment?	A. Yes, always<br><br>B. Yes, sometimes<br><br>C. Never<br><br>If Never, please go to Question 14	
13.	Why did you switch antibiotics during the course of self-treatment? (check more than one if applicable)	A. The former antibiotics did not work<br><br>B. The latter one was cheaper<br><br>C. To reduce adverse reactions	

14.	Have you ever found out that you had taken the same antibiotics with different names at the same time?	A. Yes<br><br>B. No	
15.	When did you normally stop taking antibiotics? (check more than one if applicable)	A. After a few days regardless of the outcome<br><br>B. After symptoms disappeared<br><br>C. A few days after the recovery<br><br>D. After antibiotics ran out<br><br>E. At the completion of the course<br><br>F. After consulting a doctor/pharmacist	
16.	Have you ever had any adverse reaction when you took antibiotics for self-medication?	A. Yes (specify)<br><br>B. No<br><br>If NO, please go to Question 18	
17.	What did you do for the adverse reactions? (check more than one if applicable)	A. Stopped taking antibiotics<br><br>B. Switched to another antibiotic<br><br>C. Consulted pharmacy staff<br><br>D. Consulted a doctor<br><br>E. Consulted family members/friends	
18.	Please write down the names of antibiotics you have ever taken for SELF-MEDICATION:		
19.	What do you think about self-medication with antibiotics for self-health care	A. Good practice<br><br>B. Acceptable practice<br><br>C. Not acceptable practice	
20.	Do you think you can treat common infectious diseases with antibiotics successfully by yourself?	A. Yes, I can<br><br>B. Not sure<br><br>C. No, I cannot	
